# Our Bureau of Information

**Published:** 1912-02-24

**Authors:** 


### OUR BUREAU OF INFORMATION.

### The Problem of After-Care.

One of the disheartening things connected with hospital work is that patients must go back to conditions which are more likely to hinder their cure rather than to help it. This is not a problem of poverty pure and simple. The question of means bulks largely when it is the bread-winner who has been laid up, but relapses also occur when other members of the family return from hospital. It is very largely a matter of ignorance. In hospital what the doctors have ordered has been carefully and conscientiously done. Medicine has been given to the moment, only the proper food has been allowed, and peace and quiet, if not rest, have been rigidly enjoined. The home-maker—the patient's wife or mother—does not know the importance of these things, and so the patient suffers and relapses. The institution of lady almoners who follow the patients to their homes has been of incalculable benefit in dealing with such cases, but it is not possible for them to look after cases at a great distance, and people come from far and near to our London hospitals. Moreover, their ministrations are often confined to out-patients. That out-patients who have never known the benefit of hospital *régime* specially need looking after cannot be doubted, but unless the late in-patient is of age and intelligence to insist on going on with hospital methods in the home as far as possible, he is apt to relapse to something approaching his former condition. Perhaps, if he is lucky, he may get back to the hospital, but perhaps his relations may think that if the treatment is to be of only temporary benefit it is hardly worth while renewing it. And all the suffering and trouble might be avoided by a little guidance and after-care.

Nor is it difficult to render this care in our country. Nowhere in the world is there such an organisation of district nursing as in Britain. There are very few districts which lack a nurse who could, and gladly would, keep an eye on the ex-patient, advise as far as possible, and do her best to see that the treatment which was carried on at the hospital is continued for a due length of time. What is needed is an organisation within every hospital for communicating with district nurses, and telling them what is required for each case. The communication should be sent direct to the nurse of the neighbourhood, or to the doctor who has previously had charge of the case, to make sure that it will receive due attention. This, of course, would mean an addition to the clerical staff of the hospital, and few hospitals have money to spare; but it would add greatly to the permanent efficiency of their work. The Cicely Northcote Trust does good work in this way among the out-patients of St. Thomas's Hospital, but perhaps it undertakes more in the way of what may be called absolute philanthropy. We do not suggest anything in the way of almsgiving, but only the making use of existing machinery to continue outside the hospital walls the treatment which has benefited the patients within them. The outside machinery exists, but the inside machinery is by no means universal. It would cost something to establish it, but not a great deal, and when the need is realised we have little doubt that it will be met, either by private benevolence or by some grant from special hospital funds.

### NEEDS AND HELPERS.

### HOW TO BECOME A HOSPITAL ALMONER.

"Public Health" writes: Will you kindly inform me through the medium of The Hospital from what source lady almoners are obtained? Where, if any, is the special place of training? How could such a position be obtained by an educated woman with public health qualifications?

"Public Health" should write to the Hospital Almoners' Council, Denison House, Vauxhall Bridge Road, London, S.W. She will there get full information as to how to prepare for almoners' work. The Council has been formed for the purpose of giving information and assistance to those who wish to secure appointments in this work. The demand for almoners, though growing, is still not large. "Public Health" should keep her eye upon our list of vacant appointments.

### Help for an Invalid Governess.

"Sister Elinor" writes: I am so sorry for "Borage," she is so young to be suffering from neurasthenia. Could you let her know that there is a free holiday home for governesses at "St. Athelstan," Chapel Park Road, St. Leonards-on-Sea? The lady in charge is Miss L. M. Smyth. If this is not suitable, she might get help from the "Women's Holiday Fund," 76 Denison House, Vauxhall Bridge Road.

### Laundry for Consumptives.

"Lutetia" writes: In Paris there is a laundry—founded, I believe, by Mademoiselle Chaptal—where the clothing of consumptives is washed under careful sanitary precautions. This is a great boon, especially to the poor, whose clothes are washed free, and prevents any possible infection going from the patient's clothes to those of other members of the family. Is there anything of the kind in London?

### Lady Dentists.

"Riccarda" writes: Can a woman become a properly qualified dentist in this country? Is there a demand for lady dentists?

A woman can become a fully-qualified dentist here. Women dental students can go through the necessary medical training at the London School of Medicine for Women, and special dental studies at the National Dental Hospital, Great Portland Street. They are also eligible for the diploma in dental surgery of the Royal College of Surgeons of Edinburgh. We do not think that there is any special demand for lady dentists.

### Our List of Approved Homes.

We have pleasure in adding the following to our list of Approved Homes:—

45 Clarence Square, Brighton. Principal, Sister Miriam. Surgical, medical, and chronic. Large sunny rooms. Acute cases, £5 5s. per week; chronic and convalescent, 2½ to 3 guineas per week. Children received.

### Rules for Correspondents.

Everyone who uses or wishes to help our Bureau of Information must be kind enough to observe the following rules:—

(1) Postal replies cannot be given. Exception will only be made in very special cases at the Editor's discretion.

(2) Queries or answers relating to different cases must be written, or preferably typed, on separate sheets of paper, and the writing must never be crossed.

(3) Questions and replies received not later than Wednesday will, as far as possible, be dealt with in our issue of the following Saturday week.

(4) Letters must have attached the name and address of the sender, with a pseudonym for publication if preferred.

(5) Applications referring to non-dependents where the need relates to business or employment must be accompanied by a postal order for 5s., when the requirement shall have publicity in these columns.

(6) All applicants for insertion in our list of Approved Homes must send a registration fee of five shillings. The omission of this leads to delay and gives avoidable trouble.

(7) *All communications* for this department *must be accompanied by the coupon* to be found at the bottom of the third page of the cover on the inside, and should be addressed to the Editor of the Bureau of Information, c/o The Hospital, 29 Southampton Street, Strand, London, W.C.

